# Genome-Wide Characterization of the *Botryosphaeria dothidea* GH28 Family Reveals BdGH28_3 Contributes to Virulence on Chinese Hickory

**DOI:** 10.3390/plants15162547

**Published:** 2026-08-21

**Authors:** Dong Liang, Wei Ai, Yi-Ru Jiang

**Affiliations:** College of Advanced Agricultural Sciences, Zhejiang Agriculture and Forest University, Hangzhou 311300, China

**Keywords:** Chinese hickory canker, *Botryosphaeria dothidea*, Glycosyl hydrolase family 28 (GH28), pectin-degrading enzymes

## Abstract

Chinese hickory (*Carya cathayensis* Sarg.) is an economically important tree species widely cultivated in southeastern China, where trunk canker disease caused by *Botryosphaeria dothidea* poses a serious threat to tree health and production. Pectin-degrading enzymes are important virulence-associated factors that facilitate fungal colonization and host tissue maceration, but their evolutionary diversification and functional roles in *B. dothidea* during woody host infection remain poorly understood. Comparative genomic analysis revealed lineage-specific variation in the GH28 glycoside hydrolase family among the examined Botryosphaeriaceae species, with *B. dothidea* exhibiting an expanded GH28 repertoire relative to the analyzed species. Expression analysis and functional assays revealed that *BdGH28_3* showed the highest transcript abundance during infection stage and contributed to the full virulence of *B. dothidea*. A predicted protein–protein interaction (PPI) network suggested potential associations between BdGH28_3 and other pectinolytic enzymes, including polygalacturonases, pectin lyases, and pectinesterases. Collectively, these findings identify GH28 diversification as a distinctive feature of the *B. dothidea* genome and establish *BdGH28_3* as a virulence-associated member, providing a foundation for investigating GH28-mediated pathogenicity in woody hosts.

## 1. Introduction

Chinese hickory (*Carya cathayensis* Sarg.), a nut-producing tree species belonging to the Juglandaceae family and widely cultivated in southeastern China, is economically important because of its edible kernels with high nutritional value [1]. Statistics indicate that the cultivation area of Chinese hickory trees exceeds 15,000 hectares in the Zhejiang and Anhui provinces of China [2]. However, as the cultivation area of Chinese hickory has expanded markedly over the past few decades, the incidence of trunk canker disease has become increasingly severe [3]. This disease typically manifests from March to September, beginning as small, elliptical lesions at the sites of bark infection. These lesions subsequently enlarge into sunken, elongated cankers that coalesce into extensive, black, and diffuse areas of blighted tissue [4].

*Botryosphaeria* species are known to cause canker and dieback in a variety of woody hosts, including apple, pear, grapevine, almond, citrus, plum and poplar [5]. Notably, *Botryosphaeria dothidea* is a predominant species and serves as a major causal agent of trunk canker in Chinese hickory [6]. This pathogen infects through wounds, breaches the outer bark, and colonizes the phloem, vascular cambium, and xylem, leading to cortical decay and the formation of dark cankers, which ultimately result in fruit drop and reduced yield [7]. Currently, there are few effective measures to control Chinese hickory canker, and the excessive application of fungicides has resulted in significant environmental issues [8]. Therefore, it is imperative to develop more effective and environmentally friendly control strategies. To investigate the molecular mechanism underlying the pathogenicity of *B. dothidea* in Chinese hickory, we previously reported the draft genome of *B. dothidea* BDLA16-7, the dominant pathogen isolated from trunk canker samples [9].

Carbohydrate-active enzymes (CAZymes), classified into five superfamilies [10], including glycoside hydrolases (GHs), glycosyltransferases (GTs), polysaccharide lyases (PLs), and carbohydrate esterases (CEs) and auxiliary activities (AAs), mediate the degradation of host plant cell walls and are critical for successful host colonization and fungal proliferation [11]. For example, the disruption of GH6 and GH7 hydrolases, which cleave cellulose β-1,4-glucans and weaken the primary cell wall, significantly reduces penetration efficiency and subsequent lesion formation in *Magnaporthe oryzae* [12]. In additionally, some GH10 and GH11 xylanases, exhibiting endo-β-1,4-xylanase activity, contribute to appressorium formation in *M. oryzae*, and also function as secreted effectors that interfere with host plant immunity [13,14].

Pectin is a major acidic polysaccharide found in the primary cell wall and middle lamella of plants, characterized by a backbone composed of α-1,4-linked galacturonic acid residues [15]. Its primary functions include the hydration and adhesion of plant cell walls, which contribute to the stability and flexibility of these structures [16,17,18]. Notably, pectin-degrading enzymes secreted by plant pathogens such as *Erwinia* spp. are known to cleave the pectic backbone of the plant cell wall, resulting in host cell necrosis and tissue maceration or decay [19,20]. Plant–pathogen interactions are also accompanied by dynamic host responses, in which pathogen-derived molecules can influence plant defense and disease development [21]. Furthermore, *B. dothidea* is recognized for causing fruit rot across a wide range of hosts [5], suggesting that pectin-degrading enzymes play a significant role in the pathogenicity of *B. dothidea*. Polygalacturonase (PG), a member of Glycoside Hydrolase Family 28 (GH28), catalyzes the hydrolysis of alpha-(1,4)-glycosidic linkages between galacturonic acid residues in pectin [22]. Previous studies have suggested a close association between the GH28 family and plant pathogenicity [21]. VdEPG1, a GH28 enzyme of *Verticillium dahliae*, modulates host immunity by interfering with jasmonic acid (JA) biosynthesis [23], consistent with the broader concept that microbial signals can influence plant signaling and defense responses [24]. Furthermore, this enzyme facilitates mycelial development and conidiation while enhancing the pathogen’s ability to penetrate cellophane membranes [23]. Additionally, the deletion of GH28 polygalacturonases genes *BcPG1* and *BcPG2* in *Botrytis cinerea* significantly reduced lesion expansion on tomato leaves [25].

Previous studies have characterized individual GH28 enzymes primarily in herbaceous pathogens, but how GH28 repertoires have diversified in woody plant pathogens and whether this diversification is associated with pathogenicity remain largely unresolved. Genome resources from *Botryosphaeria* and other Botryosphaeriaceae species have enabled comparative analyses of fungal genome evolution and pathogenicity [26]. However, these studies have focused mainly on genome structure, phylogenetic relationships, and broad patterns of gene content, leaving the evolution and functional relevance of expanded CAZyme families less well defined. Our previous genome sequencing of *B. dothidea* strain BDLA16-7 [9] provided a basis for examining the genetic determinants of Chinese hickory trunk canker. Based on this resource, we investigated whether the expanded GH28 repertoire of *B. dothidea* has undergone lineage-specific diversification and whether individual members are associated with infection. We therefore combined comparative genomic and evolutionary analyses with infection-stage expression profiling to characterize the GH28 family and identify infection-responsive members, followed by targeted functional analysis of *BdGH28_3* to assess its contribution to fungal virulence on Chinese hickory.

## 2. Results

### 2.1. Comparative Analysis Reveals Enrichment of the GH28 CAZyme Family in B. dothidea

**Figure 1:**
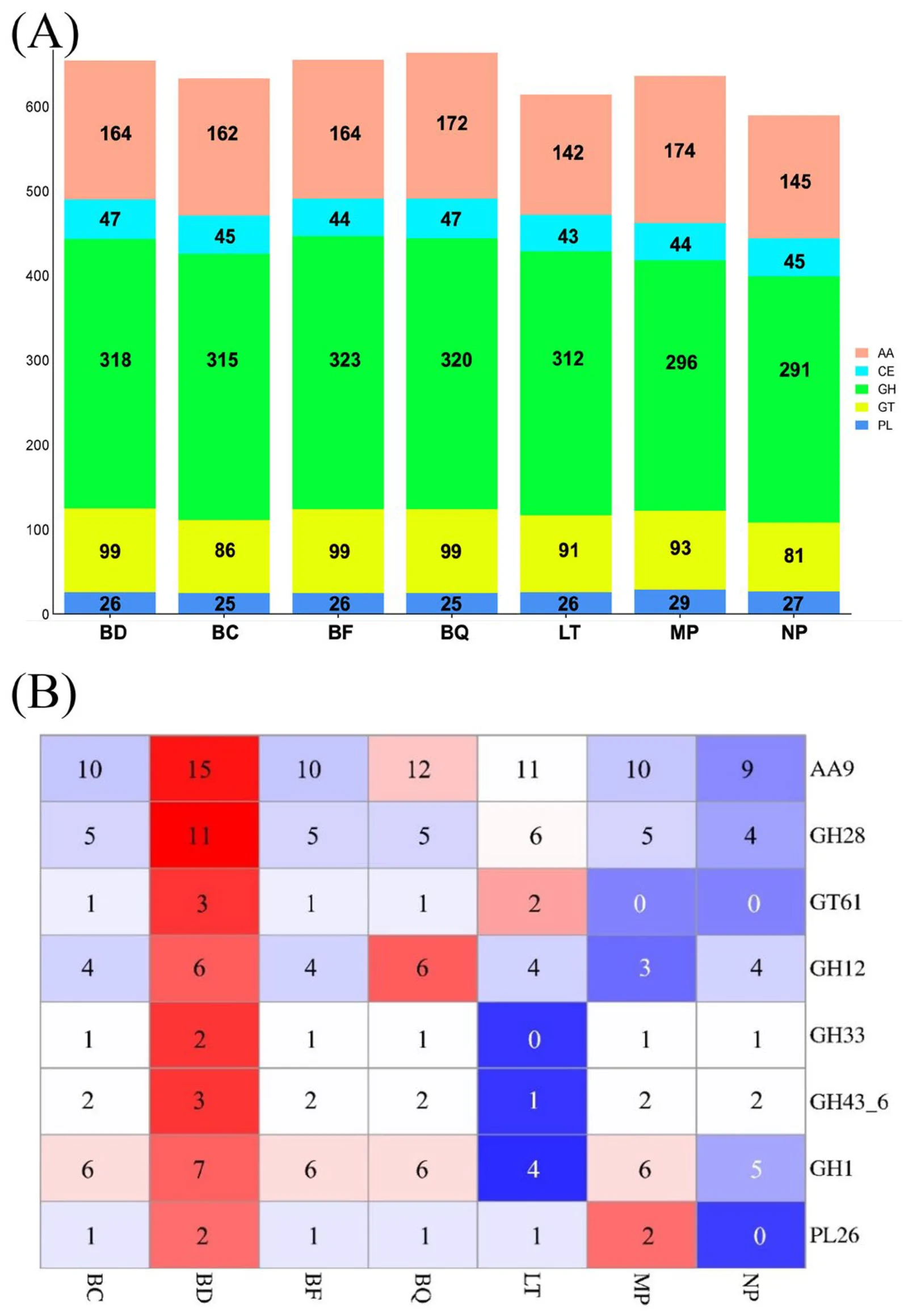
Distribution of carbohydrate-active enzyme (CAZyme) classes and enriched CAZy families across seven Botryosphaeriaceae species. (**A**) Number of proteins assigned to the five major CAZyme classes, including auxiliary activities (AAs), carbohydrate esterases (CEs), glycoside hydrolases (GHs), glycosyltransferases (GTs), and polysaccharide lyases (PLs). (**B**) Comparison of CAZy families enriched in *B. dothidea* based on Fisher’s exact test and odds ratio (OR) analysis. *p*-values were adjusted for multiple testing using the Benjamini–Hochberg procedure, and an adjusted *p* < 0.05 was considered statistically significant. The heatmap indicates the number of proteins assigned to individual CAZy families across the seven species. Species abbreviations: BD, *Botryosphaeria dothidea*; BC, *Botryosphaeria cortices*; BF, *Botryosphaeria fabicerciana*; BQ, *Botryosphaeria qingyuanensis*; LT, *Lasiodiplodia theobromae*; MP, *Macrophomina phaseolina*; NP, *Neofusicoccum parvum*.

The identified CAZymes were classified into five major groups, including glycoside hydrolases (GHs), auxiliary activities (AAs), glycosyltransferases (GTs), carbohydrate esterases (CEs), and polysaccharide lyases (PLs). As shown in Figure 1A, the overall composition and abundance of CAZyme categories were comparable among the seven Botryosphaeriaceae species. In *B. dothidea*, 318 GHs, 163 AAs, 99 GTs, 47 CEs, and 26 PLs were identified, which fell within the ranges observed across the other six species (291–323 GHs, 142–174 AAs, 81–99 GTs, 43–47 CEs, and 25–29 PLs).

To identify CAZyme families specifically enriched in *B. dothidea*, gene numbers assigned to individual CAZy families were compared using Fisher’s exact test and Odds Ratio (OR) analysis. As shown in Figure 1B, several CAZy families displayed higher representation in *B. dothidea* (OR > 1), including GH28, GT61, AA9, GH1, GH12, GH33, GH43_6, and PL26. After Benjamini–Hochberg correction, GH28 was the only family showing statistically significant enrichment (adjusted *p* = 0.028). Consistently, the gene count heatmap revealed that *B. dothidea* possessed 11 GH28 proteins, whereas the other six Botryosphaeriaceae species contained only five to six GH28 members (Figure 1B).

### 2.2. Evolutionary Analysis of the GH28 Family

**Figure 2:**
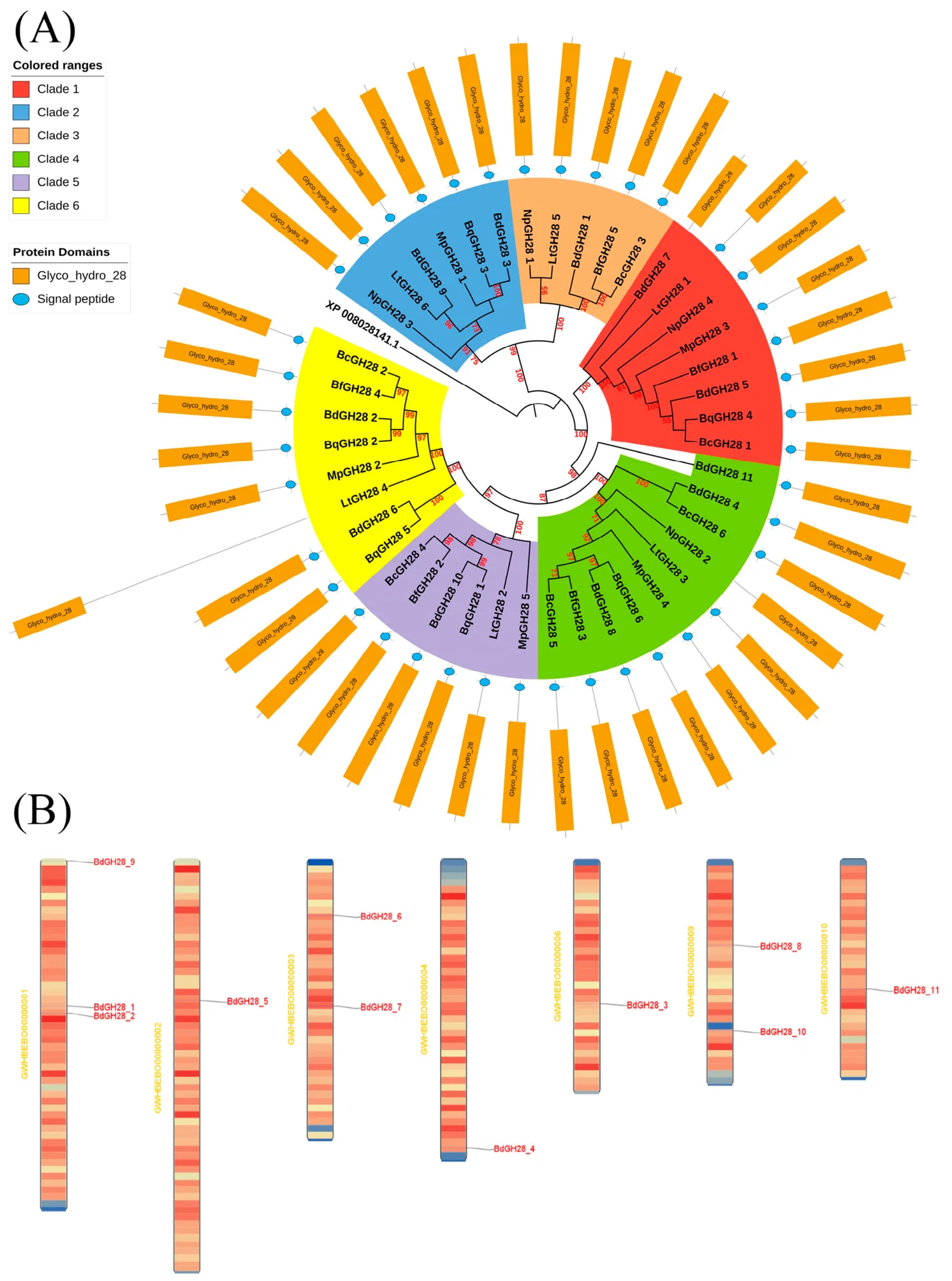
Phylogenetic relationships and genomic distribution of GH28 genes across seven Botryosphaeriaceae species. (**A**) Phylogenic tree of GH28 proteins identified in seven *Botryosphaeriaceae* species. Maximum likelihood tree, with 1000 bootstraps (values displayed per branch). Signal peptides and domain architectures were shown alongside the phylogenetic tree. (**B**) Genomic distribution of the BdGH28 genes across the *B. dothidea* genome. Color intensity indicates gene density across each genomic region.

To elucidate the evolutionary relationships within the GH28 family, an maximum likelihood (ML) phylogenetic tree was constructed based on 41 GH28 sequences, comprising 11 from *B. dothidea* (BD), 5 from *B. cortices* (BC), 5 from *B. fabicerciana* (BF), 5 from B. qingyuanensis (BQ), 6 from *L. theobromae* (LT), 5 from *M. phaseolina* (MP) and 4 from *N. parvum* (NP) (Table S1). The results indicated that these 41 GH28s were categorized into six distinct clades (Clade 1–6) (Figure 2A). Clade 1 was composed of eight members: two BdGH28s and one from each of BC, BQ, BF, LT, MP, and NP. Clade 2 comprised eight GH28s, with one representative from each of BD, BC, BF, LT, and NP. Clade 3 included seven GH28s, featuring two BdGH28s and one from each of BQ, LT, NP, and MP. Clade 5 contained six GH28s, with one from each of BD, BC, BQ, BF, LT, and MP. Clade 6 also encompassed eight GH28s, which included two BdGH28s and one from each of BC, BQ, BF, LT, and MP. Notably, Clade 4 consisted of ten GH28 proteins: three BdGH28s, two BcGH28s, and one from each of BQ, BF, NP, LT, and MP. Although *B. dothidea* contained additional GH28 members in Clades 1, 3, 4, and 6 compared with several other Botryosphaeriaceae species, these members were phylogenetically dispersed without lineage-specific clustering. BdGH28 members were distributed across Clades 1, 3, 4, and 6 rather than forming a single species-specific cluster.

**Figure 3:**
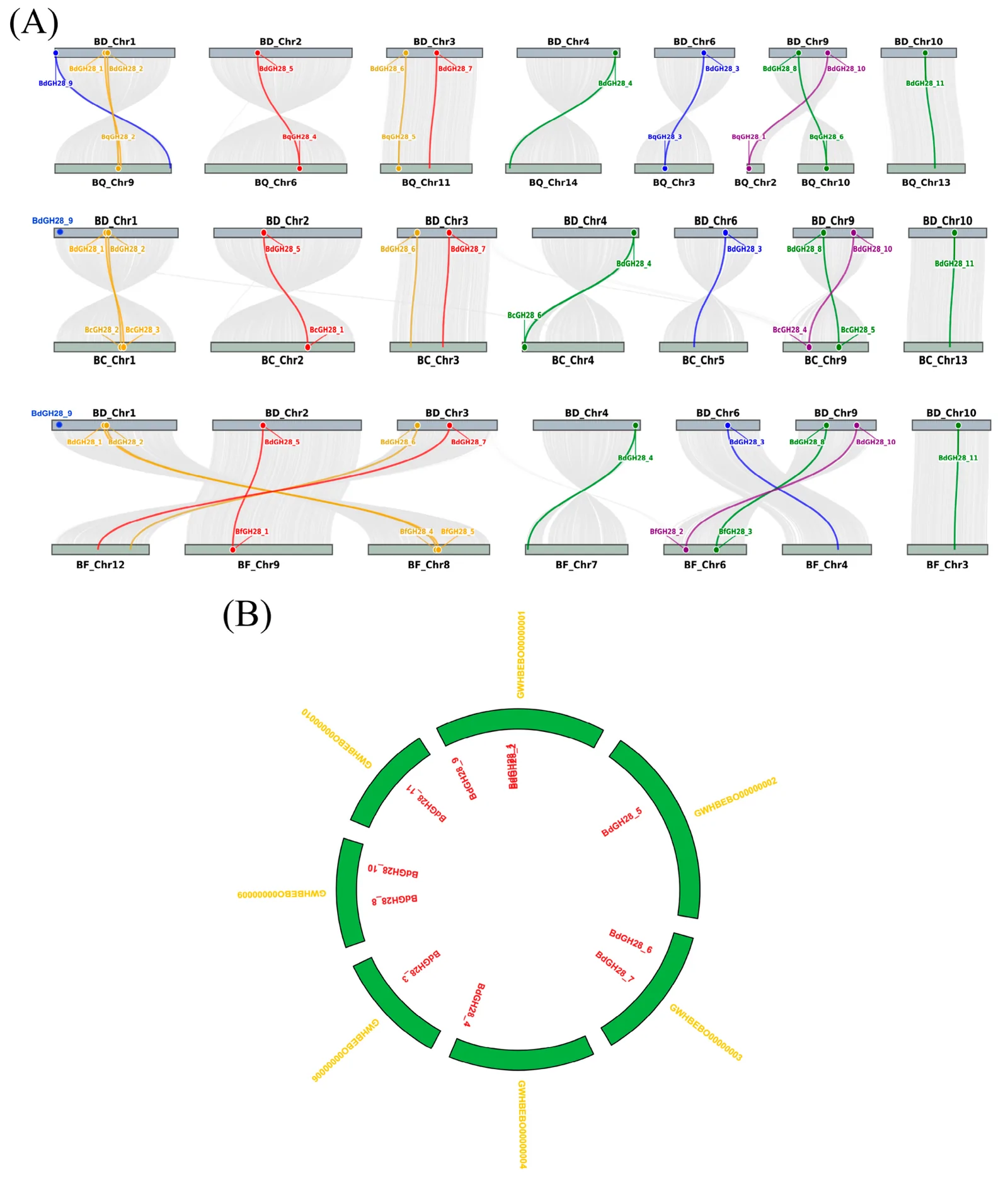
Synteny analysis of GH28 genes. (**A**) Interspecies syntenic relationships of GH28 genes in *B. dothidea*, *B. cortices*, *B. qingyuanensis* and *B. fabicerciana*. Red, blue, orange, green, purple, and yellow circles indicate GH28 genes assigned to clades 1–6 of the phylogenetic tree, respectively. (**B**) Intraspecies syntenic relationships among GH28 genes in the *B. dothidea* genome.

Signal peptides and transmembrane domains were predicted for all BdGH28 proteins. As shown in Figure 2A, all BdGH28 proteins lacked transmembrane domains, whereas signal peptides were predicted for all members except BdGH28_5 and BdGH28_7, both of which clustered in clade 1. Domain architecture analysis indicated that the Glyco_hydro_28 domain of most BdGH28 proteins resides in the C-terminal region. Only BdGH28_1 in clade 2 represents an exception, as its Glyco_hydro_28 domain is located in the central region of the protein. To investigate the evolutionary conservation of GH28 loci among Botryosphaeriaceae species, we analyzed inter-species collinearity (Figure 3A). The strongest syntenic conservation was observed between *B. dothidea* and *B. qingyuanensis*, with *BdGH28_3/9* and *BdGH28_4/8* showing conserved syntenic relationships with *BqGH28_3* and *BqGH28_6*, respectively. However, several *BdGH28* genes (*BdGH28_3/4/6/7/11*) lacked detectable collinear homologs in BC and BF, indicating differences in GH28 gene retention patterns among these species. However, no collinear relationships were detected among *BdGH28* genes within the *B. dothidea* genome (Figure 3B).

### 2.3. Genomic Location, Conserved Motifs, and Structural Features of BdGH28s

The chromosomal mapping revealed an uneven distribution of all 11 *BdGH28s* across seven scaffolds (Figure 2B). Specifically, *BdGH28_1*, _2, and _9 are located on scaffold GWHBEBO00000001, while *BdGH28_6* and _7 are found on scaffold GWHBEBO00000003. Additionally, *BdGH28_8* and _10 are situated on scaffold GWHBEBO00000009, with the remaining four members dispersed across scaffolds GWHBEBO00000002, GWHBEBO00000004, GWHBEBO00000006, and GWHBEBO00000010. Most *BdGH28* genes were located in gene-rich regions and were distributed across different scaffolds without adjacent gene arrangements.

**Figure 4:**
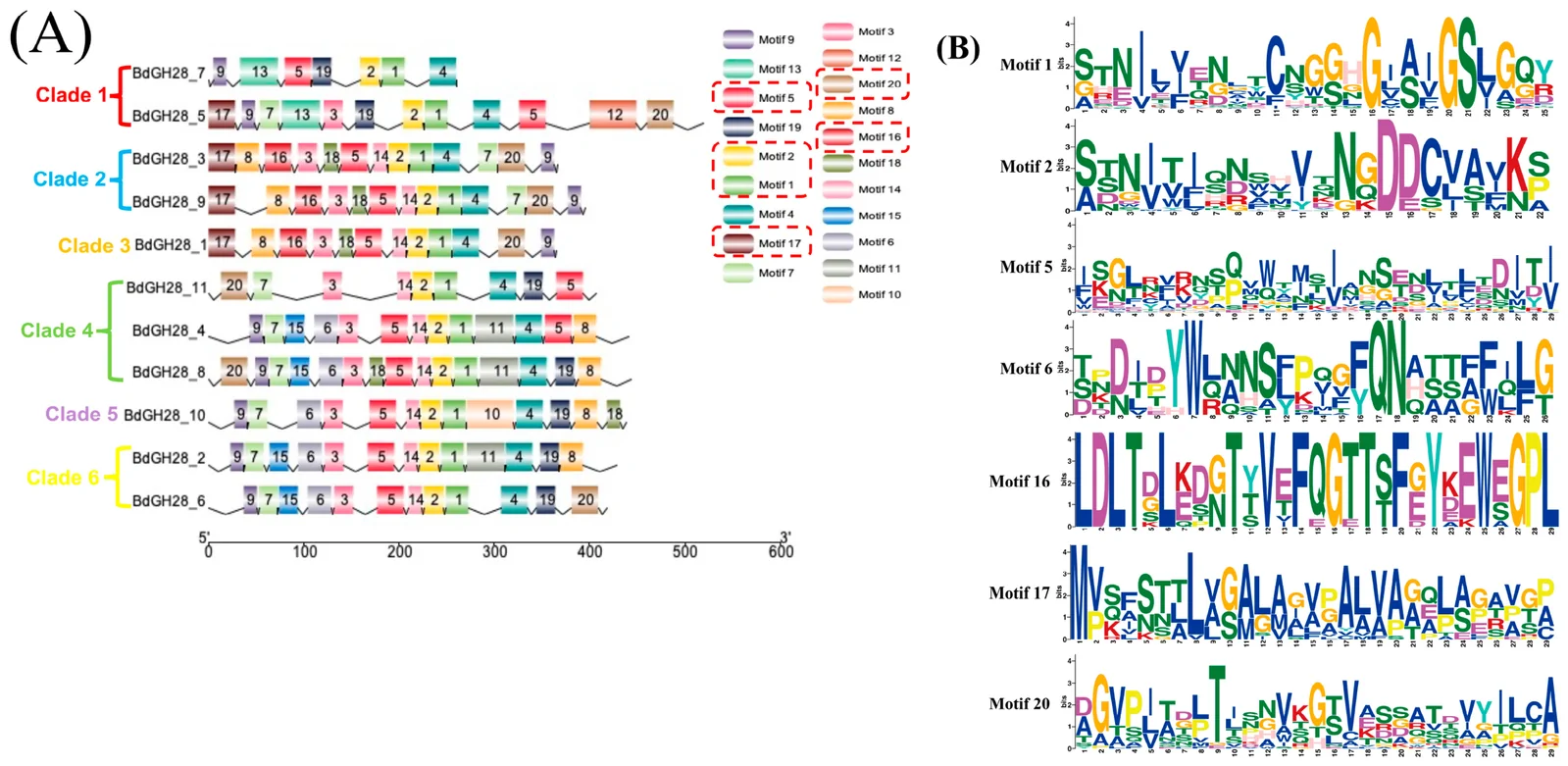
Architecture of conserved motifs and gene structures of the GH28 family in *B. dothidea*. Boxes with different colors represented different conserved motifs. (**A**) Conserved motif architecture of the 11 BdGH28 proteins, which are classified into six phylogenetic clades. The 20 conserved motifs identified in the BdGH28 family are shown as numbered, color-coded boxes, with their positions and relative lengths indicated along each protein sequence. (**B**) Sequence logos of seven representative conserved motifs (Motifs 1, 2, 5, 6, 16, 17, and 20) showing the amino acid composition and degree of conservation at each position. Letter height reflects the relative frequency of each amino acid within the corresponding motif.

A total of twenty conserved motifs were identified among the eleven BdGH28 proteins using the MEME Suite with default parameters, with a maximum of 20 motifs specified for motif discovery. Motifs 1, 2 and 5 were present in all BdGH28 proteins, indicating a high level of conservation within the GH28 family (Figure 4A). Furthermore, the previously reported conserved catalytic-associated sites, CGPGHGIS, SPNTDGI, and GDDC, were found within motifs 1 and 2 (Figure 4B). The distribution and positioning of motifs 7 and 9 exhibited relative conservativeness among the internal members of the clade. In contrast, motifs 16, 17, and 20 were primarily restricted to members of clades 1, 2, 3 and 4, while BdGH28s containing motif 6 were predominantly clustered within clades 4, 5, and 6. Motif composition differed among phylogenetic clades, with motifs 16, 17, and 20 mainly detected in Clades 1–4, whereas motif 6 was enriched in Clades 4–6.

### 2.4. Tissue Expression Profiles of BdGH28 Genes During B. dothidea Infection

**Figure 5:**
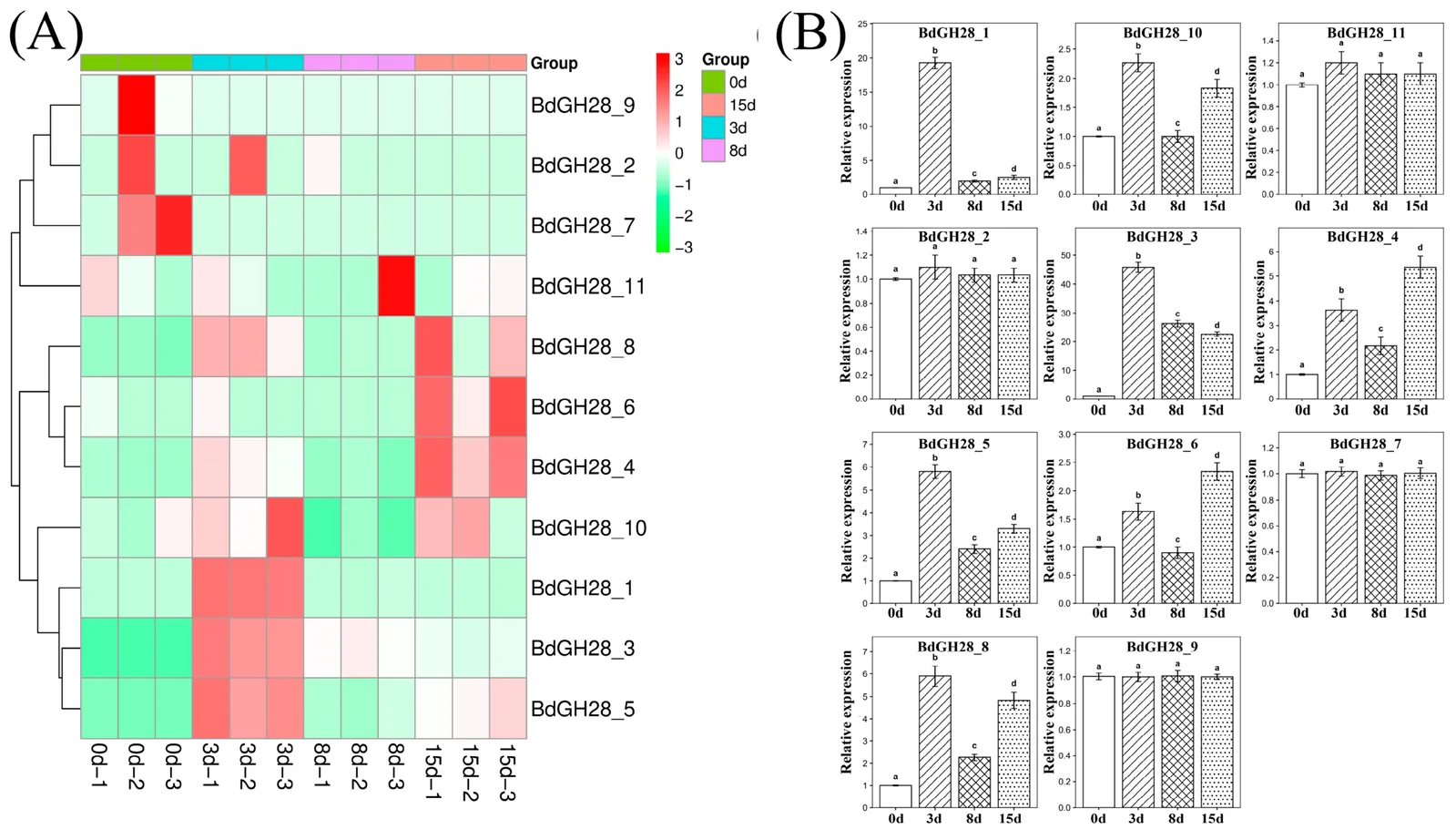
Infection-stage expression profiles of BdGH28 genes during *B. dothidea* infection of Chinese hickory. (**A**) RNA-seq expression profiles of *BdGH28* genes during interaction with Chinese hickory at 0, 3, 8, and 15 days post-inoculation (dpi). Expression levels are shown as log2-transformed fold changes relative to the control. Differential expression was determined using DESeq2, with |log2FC| ≥ 1 and a Benjamini–Hochberg adjusted *p*-value (FDR) < 0.05 considered significant. (**B**) Quantitative reverse transcription PCR (qRT-PCR) validation of selected *BdGH28* genes during infection. Expression levels at 0 dpi were used as the reference and normalized to 1.0. The β-tubulin gene of *B. dothidea* was used as the internal reference gene. Data are presented as mean ± SD from three independent biological replicates. Differences among time points for each gene were evaluated using one-way ANOVA followed by Tukey’s multiple-comparison test. Different lowercase letters indicate significant differences among time points at *p* < 0.05.

To investigate the expression of BdGH28 genes during the infection process of *B. dothidea*, we analyzed transcript levels using our previously reported RNA-seq data (NCBI SRA Accession: PRJNA1137604) [27]. In this study, detached branches of Chinese hickory were inoculated with the wild-type *B. dothidea* strain BDLA16-7 and incubated at 25 °C. As illustrated in Figure 5A, tissues surrounding the inoculation sites were harvested at 3, 8, and 15 days post-inoculation (dpi) for RNA-Seq sequencing and analysis. *BdGH28_1*, *BdGH28_3*, *BdGH28_5*, and *BdGH28_10* were significantly up-regulated at 3 dpi based on DESeq2 analysis (FDR < 0.05), whereas *BdGH28_4*, *BdGH28_6*, *BdGH28_8* exhibited peak expression at 15 dpi. Conversely, *BdGH28_2*, *BdGH28_7* and *BdGH28_9* exhibited down-regulation throughout the infection process. *BdGH28* genes exhibited distinct infection-associated expression patterns. Using the same sampling procedure described above, quantitative reverse transcription polymerase chain reaction (qRT-PCR) assays were performed to validate the expression patterns of these *BdGH28* genes. As illustrated in Figure 5B, *BdGH28_1*, *BdGH28_3* and *BdGH28_5* were consistently up-regulated at 3 dpi. Moreover, *BdGH28_4* and *BdGH28_8* exhibited continuously increased expression levels. In addition, *BdGH28_2*, *BdGH28_7* and *BdGH28_9* remained relatively stable compared with that at 0 dpi (control samples). Overall, a comparison of qRT-PCR and RNA-Seq data revealed consistent expression patterns for most *BdGH28* genes. Among the 11 *BdGH28* genes, *BdGH28_3* showed the highest transcript abundance at 3 dpi and was therefore selected for subsequent functional characterization.

### 2.5. BdGH28_3 Is Required for Full Virulence of B. dothidea on Chinese Hickory

**Figure 6:**
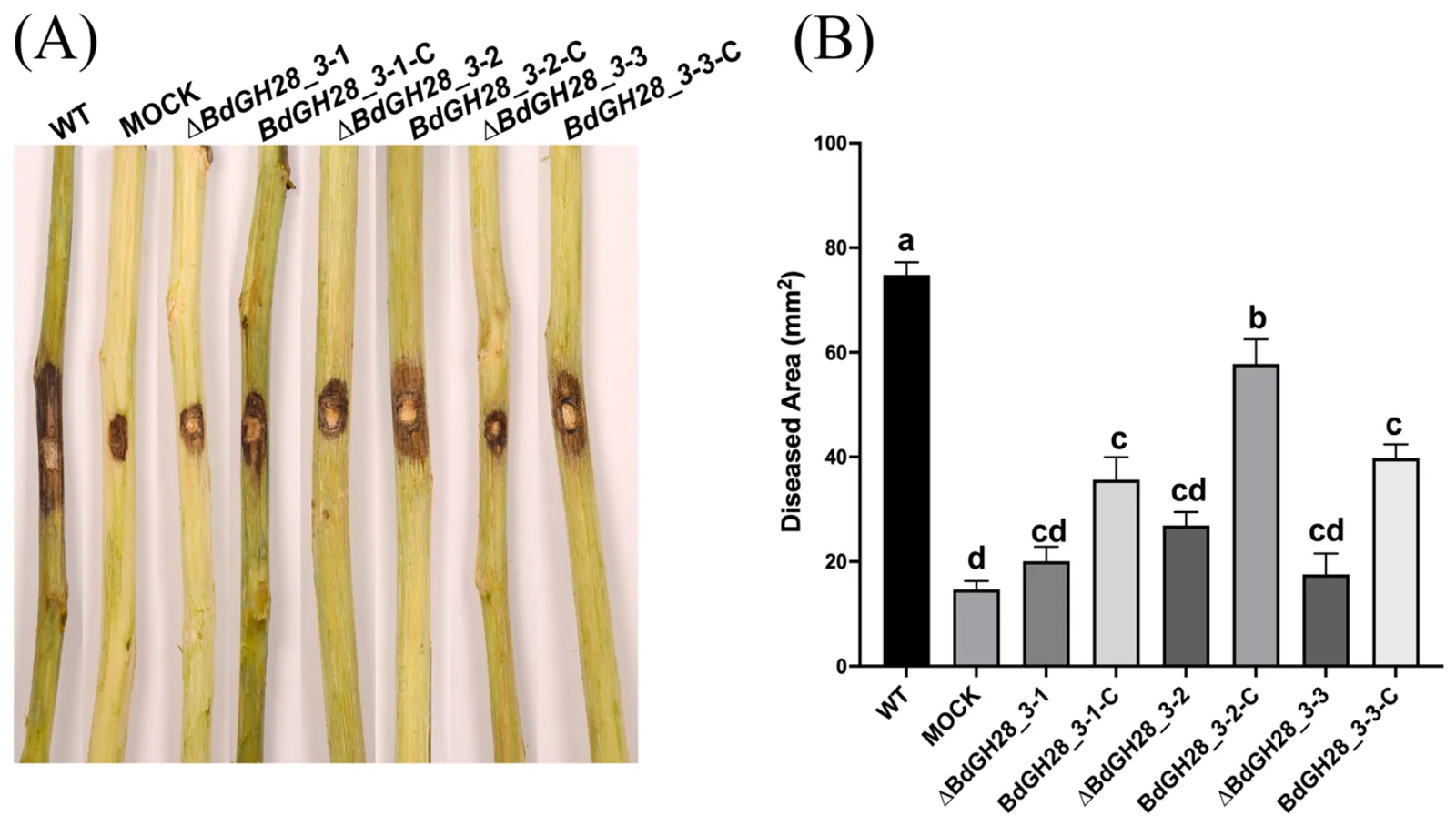
Effect of *BdGH28_3* deletion on the virulence of *B. dothidea* on Chinese hickory. (**A**) Representative disease symptoms caused by the wild-type strain, three independent Δ*BdGH28_3* deletion mutants, and their corresponding complemented strains on detached Chinese hickory branches at 15 dpi. (**B**) Quantification of lesion area at 15 dpi measured directly from the necrotic tissue using ImageJ2. Lesion area (mm^2^) at 15 dpi was used as a quantitative measure of disease severity for comparison of fungal virulence among strains. Data are presented as mean ± SD from three independent detached branches for each fungal strain. Different lowercase letters indicate statistically significant differences among fungal strains according to one-way ANOVA followed by Tukey’s multiple-comparison test (*p* < 0.05).

Given the strong induction of BdGH28_3 during infection, we investigated its contribution to virulence by generating three independent *BdGH28_3* deletion mutants (Δ*BdGH28_3*-1, Δ*BdGH28_3*-2, and Δ*BdGH28_3*-3) in the *B. dothidea* wild-type strain BDLA16-7. To verify that any virulence defect was specifically associated with *BdGH28_3* deletion, the full-length *BdGH28_3* gene was reintroduced into the deletion mutants for complementation analysis. Notably, 15 dpi was selected as the endpoint for quantitative assessment of lesion development. At this time point, the wild-type strain caused extensive necrotic lesions surrounding the inoculation sites on detached Chinese hickory branches, whereas all three Δ*BdGH28_3* deletion mutants produced visibly smaller lesions (Figure 6A). Complementation with *BdGH28_3* (Δ*BdGH28_3*-1-C, Δ*BdGH28_3*-2-C, and Δ*BdGH28_3*-3-C) increased lesion development relative to the corresponding deletion mutants, although the complemented strains did not fully reproduce the wild-type phenotype (Figure 6A). Consistent with the representative disease symptoms shown in Figure 6A, quantitative measurement of lesion area using ImageGP version 2 showed that the three Δ*BdGH28_3* deletion mutants produced lesions of approximately 20–25 mm^2^ at 15 dpi, compared with nearly 80 mm^2^ for the wild-type strain (Figure 6B). Complementation significantly increased lesion area relative to the corresponding deletion mutants (one-way ANOVA followed by Tukey’s multiple-comparison test, *p* < 0.05), although lesion development remained lower than that of the wild-type strain (Figure 6B). These results demonstrate that *BdGH28_3* contributes to the full virulence of *B. dothidea* on Chinese hickory.

### 2.6. Predicted Protein–Protein Interaction (PPI) Network of BdGH28_3

**Figure 7:**
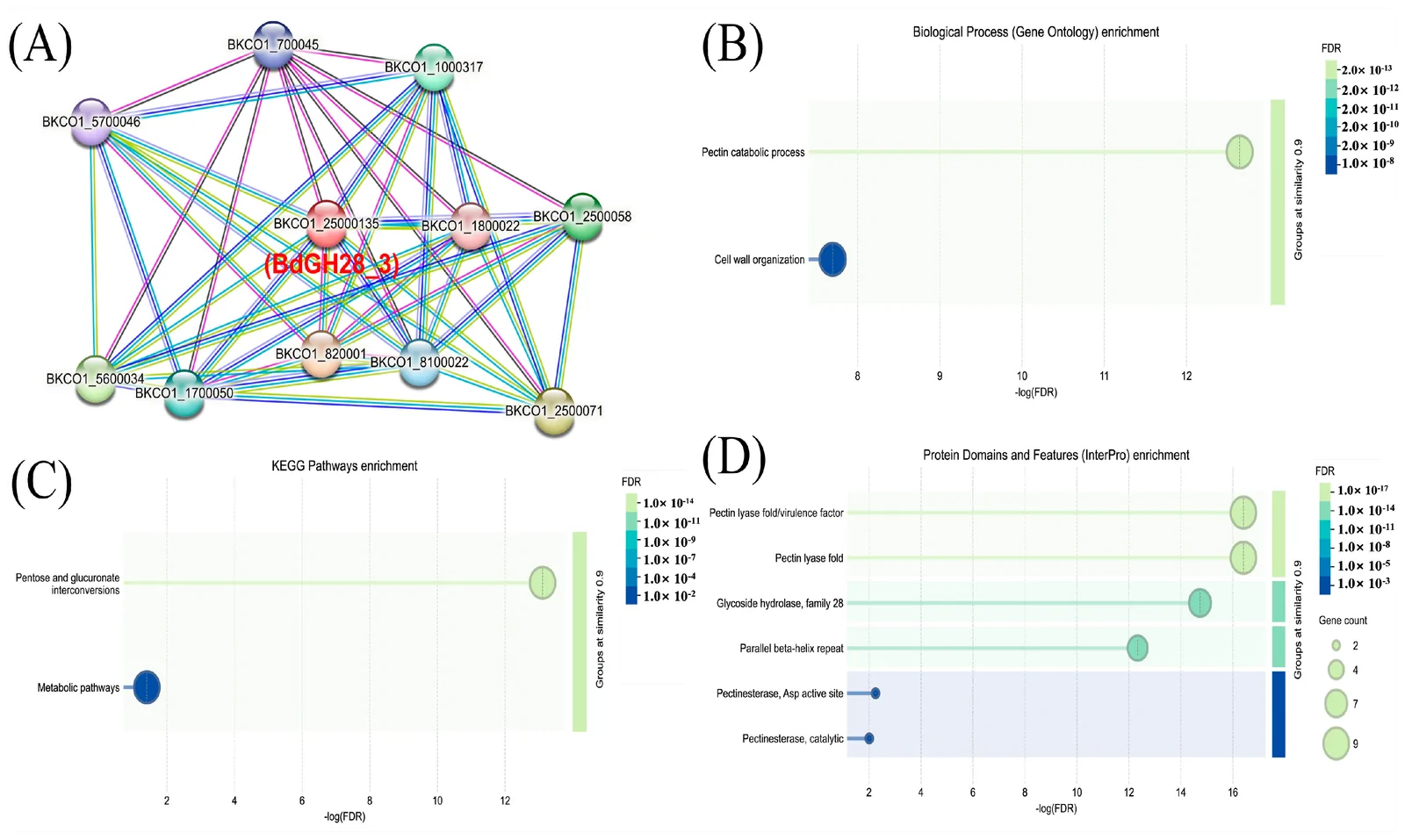
Predicted functional association network and enrichment analysis of BdGH28_3-associated proteins. (**A**) Predicted protein–protein association network of BdGH28_3 generated using the STRING v11.5 database (https://string-db.org/, 10 Match 2026). Nodes represent BdGH28_3 and its predicted functionally associated proteins, and edges represent different types of supporting evidence. (**B**–**D**) Gene Ontology (GO), KEGG pathway, and protein domain enrichment analyses of the BdGH28_3-associated proteins, respectively. Enriched terms were identified using the default enrichment analysis implemented in STRING, with an FDR < 0.05 considered statistically significant.

To explore potential functional associations of BdGH28_3, a predicted protein association network was constructed using the STRING database (Figure 7A). We identified ten proteins predicted to be functionally associated with BdGH28_3 (Table S2), including other members of the GH28 family, endo-xylogalacturonan hydrolase, pectin lyase-like protein, and pectinesterase. Furthermore, we performed functional enrichment analysis of the BdGH28_3-interacting proteins as displayed by Figure 7B–D. The enrichment analysis of Gene Ontology (GO) revealed that the predicted associated proteins were enriched in pectin degradation-related functions. Consistently, domain analysis indicated significant enrichment of typical pectin-degrading domains, including the ‘pectin lyase fold’, ‘glycoside hydrolase family 28’ and ‘pectinesterase’. KEGG analysis showed enrichment of the pentose and glucuronate interconversion pathway. GO, protein domain, and KEGG enrichment analyses consistently associated the predicted interaction network with pectin degradation and the pentose and glucuronate interconversion pathway.

## 3. Discussion

*B. dothidea* is a widespread woody plant pathogen associated with stem canker, fruit rot, dieback, and other economically important diseases across diverse hosts [5]. In Chinese hickory, infection primarily occurs in bark tissues and subsequently develops into expanding trunk cankers, causing substantial damage to tree health and production [3]. Although carbohydrate-active enzymes (CAZymes) are recognized as important components of fungal pathogenicity by modifying host cell wall polymers and facilitating tissue colonization [11], several CAZyme-encoding genes in Botryosphaeriaceae species have also been implicated in host infection [26]. However, the evolutionary diversification and functional contribution of individual CAZyme families in woody plant pathogens remain poorly understood. Comparative genomic analysis identified GH28 as the only significantly enriched CAZyme family in *B. dothidea* among the seven Botryosphaeriaceae species examined. Infection-stage expression profiling further identified *BdGH28_3* as a strongly induced family member, and targeted deletion demonstrated that it contributes to full virulence on Chinese hickory. These findings establish GH28 diversification as a prominent feature of the *B. dothidea* CAZyme repertoire and provide a framework for understanding how pectin-associated enzymes contribute to infection by woody plant pathogens.

Pectin is a major component of the plant primary cell wall and middle lamella, where it contributes to cell adhesion, wall integrity, and regulation of plant defense responses [18,28]. During infection, many phytopathogenic fungi produce diverse pectin-modifying enzymes to alter host cell wall architecture and facilitate nutrient acquisition and tissue colonization [29,30]. The GH28 family contains several classes of pectin-degrading enzymes, including polygalacturonases, rhamnogalacturonases, and xylogalacturonases, which target different structural components of pectic polysaccharides [31,32]. Functional studies in herbaceous pathosystems have demonstrated that individual GH28 members can contribute to fungal virulence. For example, *V. dahliae VdEPG1* enhances fungal colonization by promoting disease development and modulating host immune responses [24], while *BcPG1* and *BcPG2* contribute to lesion expansion and host penetration in *Botrytis cinerea* [25,33]. However, whether GH28 diversification contributes to the pathogenic adaptation of woody plant pathogens has remained unclear. Our comparative analysis showed that *B. dothidea* contains eleven GH28 genes, exceeding the copy number observed in the other Botryosphaeriaceae genomes examined here. The expanded GH28 repertoire therefore represents a distinctive genomic feature of *B. dothidea* and raises the possibility that diversification of pectin-associated enzymes may contribute to its interaction with woody hosts.

The evolutionary origin of the expanded GH28 repertoire in *B. dothidea* remains unresolved but can be partially inferred from comparative genomic patterns. The GH28 proteins analyzed from seven Botryosphaeriaceae species were distributed across six phylogenetic clades, and BdGH28 members were dispersed among multiple clades rather than forming a species-specific cluster. In addition, synteny analysis revealed conserved genomic relationships between several *B. dothidea* and *B. qingyuanensis* GH28 loci, whereas other GH28 members lacked detectable orthologous relationships in more distantly related species. Importantly, no evidence of recent tandem duplication or local expansion was observed within the *B. dothidea* genome. Although the limited number of representative Botryosphaeriaceae genomes included here does not allow reconstruction of the complete evolutionary history of GH28 genes, these observations are consistent with a model in which GH28 family diversity was shaped by differential retention, loss, and functional divergence among Botryosphaeriaceae lineages. Broader comparative analyses involving additional woody plant pathogens will be required to determine how frequently such evolutionary patterns occur.

Expansion and diversification of pectin-degrading enzymes have been associated with host adaptation in several plant-associated microbes. In *Sclerotinia sclerotiorum*, extensive expansion of GH28 genes has been proposed to enhance pectin utilization during host tissue maceration and nutrient acquisition [34]. Similarly, diversification of polygalacturonase genes has been linked to the broad host range of *Phytophthora cinnamomi* [35]. Because pectin composition varies considerably among plant species and tissues [18,36], diversification of pectin-targeting enzymes may provide pathogens with greater flexibility in responding to different host environments. Consistent with this concept, *B. cinerea* differentially expresses individual GH28 genes during infection of distinct plant tissues, indicating functional specialization within this enzyme family [37]. In *B. dothidea*, the divergent expression patterns of BdGH28 members during Chinese hickory infection suggest that individual GH28 genes may also have acquired distinct infection-associated functions rather than acting as redundant copies.

The potential relevance of GH28 diversification may be particularly pronounced in woody hosts, where bark and cambial tissues represent complex structural barriers enriched in pectic polysaccharides. Pectin accounts for a substantial proportion of the cell wall matrix in woody plants, including approximately 13% of the inner bark dry weight in *Picea abies* [38] and 11–13% of cell wall polysaccharides in the cambium–inner bark region of Populus species [39,40]. Similarly, pectin and arabinogalactan are major matrix components of Salix bark tissues [41]. Previous pathological studies have shown that *B. dothidea* commonly colonizes bark-associated tissues in peach [42], *Helwingia chinensis* [43], and apple [44]. Together with the genomic expansion observed here, these ecological characteristics suggest that diversification of GH28 enzymes may provide *B. dothidea* with an expanded enzymatic repertoire for modifying pectin-rich woody tissues. However, whether individual BdGH28 proteins exhibit substrate preferences toward specific host pectic polymers remains to be determined through biochemical characterization.

The structural analysis of BdGH28 proteins revealed both conserved features and potential sources of functional divergence. Most BdGH28 members contain predicted signal peptides and lack transmembrane domains, supporting their potential secretion into the host apoplast. The conserved Glyco_hydro_28 catalytic domain and characteristic GH28-associated motifs, including CGPGHGIS, SPNTDGI, and GDDC, were retained among all BdGH28 proteins, indicating conservation of the catalytic framework [45]. In contrast, several motifs showed clade-associated distributions, suggesting that diversification has occurred outside the conserved catalytic regions. Similar patterns have been observed in the GH28 family of *Aspergillus niger*, where lineage-specific sequence features were proposed to influence substrate interaction and enzymatic properties [46]. Although the functional significance of these variable motifs remains unknown in *B. dothidea*, they represent candidate regions for future investigations into substrate specificity and enzyme activity.

Infection-stage transcriptional analysis further revealed temporal differentiation among *BdGH28* genes during host colonization. Several members, including *BdGH28_1*, *BdGH28_3*, *BdGH28_5*, and *BdGH28_10*, showed strong induction during early infection, whereas *BdGH28_4*, *BdGH28_6*, and *BdGH28_8* reached maximal expression at later stages. These distinct expression patterns indicate that GH28 members may contribute differently throughout disease progression. Among the identified genes, *BdGH28_3* displayed the strongest early induction and was therefore selected for functional characterization.

Deletion of *BdGH28_3* resulted in a significant reduction in lesion development (one-way ANOVA followed by Tukey’s multiple-comparison test, *p* < 0.05) on Chinese hickory, whereas complementation partially restored lesion development relative to the deletion mutants, although the complemented strains did not reach the wild-type level. These results demonstrate that BdGH28_3 contributes to the virulence of *B. dothidea*. Similar contributions of individual GH28 proteins to fungal pathogenicity have been reported for *BcPG1*, *BcPG2*, and *VdEPG1* [24,25,33]. However, because the enzymatic activity and substrate specificity of BdGH28_3 were not directly examined in this study, its precise biochemical function remains to be established. The predicted secretion signal, conserved GH28 domain, infection-associated expression pattern, and requirement for full virulence are consistent with a potential role for BdGH28_3 in extracellular processes associated with host colonization. Moreover, microbial-derived signals and molecules can influence plant signaling and defense responses, providing a potential interface through which pathogen-secreted factors may affect host–microbe interactions [23].

The predicted protein association network provides additional clues regarding the potential functional context of BdGH28_3. Candidate-associated proteins included other GH28 members as well as enzymes involved in pectin modification, such as endo-xylogalacturonan hydrolases, pectin lyase-like proteins, and pectinesterases. Enrichment analyses further linked these predicted associations with pectin-related metabolic processes and pentose and glucuronate interconversion pathways. Because these relationships were generated computationally, experimental validation will be necessary to determine whether these proteins physically interact or function within the same extracellular enzyme system. Nevertheless, the predicted network supports the possibility that BdGH28_3 operates within a broader set of cell-wall-modifying enzymes during infection rather than functioning independently.

## 4. Conclusions

This study identified GH28 family expansion as a distinctive feature of the CAZyme repertoire of *B. dothidea* through comparative genomic analyses with related Botryosphaeriaceae species. Phylogenetic and syntenic analyses suggest that the current GH28 repertoire has been shaped by evolutionary diversification and differential retention of GH28 members among Botryosphaeriaceae lineages, although additional genome sampling will be required to resolve its evolutionary history. Integrated transcriptomic and genetic analyses further demonstrated that BdGH28_3 is an infection-associated gene required for full virulence of *B. dothidea* on Chinese hickory. Together, these findings highlight the importance of CAZyme diversification in fungal adaptation to woody hosts and provide a foundation for future investigations into the molecular mechanisms underlying *B. dothidea* pathogenicity and disease management strategies.

## 5. Materials and Methods

### 5.1. Genome Retrieval and Fungal Growth Maintenance

The genomes of *B. dothidea* strain BDLA16-7 (BDLA), *B. cortices* strain 16-35 (BCTK), *B. qingyuanensis* strain 16-30 (BQTK), and *B. fabicerciana* strain 18-2 (BFLG), which were originally isolated from Chinese hickory (*Carya cathayensis*) trunk canker samples, were obtained from our previous study [9]. In addition, genome assemblies and annotations of *Macrophomina phaseolina*, *Neofusicoccum parvum* and *Lasiodiplodia theobromae* were retrieved from the National Center for Biotechnology Information (NCBI) and Joint Genome Institute (JGI) genome databases. The wild-type *B. dothidea* strain BDLA16-7 was used as a background strain for the generation of *BdGH28_3* deletion mutants and pathogenicity assays. BDLA16-7 was maintained on potato dextrose agar (PDA) plates at 25 °C, and 10-day-old mycelia were collected for subsequent experiments.

### 5.2. Annotation of the CAZymes

CAZymes-encoding genes were predicted using dbCAN2 meta server for automated carbohydrate-active enzyme annotation [47]. Briefly, hidden Markov models for all CAZy families were retrieved from the carbohydrate-active enzymes (CAZy) database to search the predicted proteomes of aforementioned fungi. Transmembrane domain and secretory signal peptides were identified using the SignalP v5.0 (https://services.healthtech.dtu.dk/services/SignalP-5.0/, 16 May 2026) and TMHMM server v2.0 (https://services.healthtech.dtu.dk/services/TMHMM-2.0/, 16 May 2026), respectively. Domain architectures were further characterized by searching the Pfam v38.1 database (www.ebi.ac.uk/interpro). For CAZy families with diverse activity, specific enzyme activities were annotated via BLASTP searches against verified proteins in the ExPASy database (https://www.expasy.org/, 20 May 2026).

### 5.3. Statistical Enrichment Analysis of CAZy Subfamilies

For CAZy family enrichment analysis, Fisher’s exact test was used to compare the representation of individual CAZy families in *B. dothidea* with those in the other Botryosphaeriaceae species. *p*-values were adjusted for multiple testing using the Benjamini–Hochberg procedure, and an adjusted *p* < 0.05 was considered statistically significant.

### 5.4. Phylogenetic Analysis of the GH28 Family

Protein sequences were aligned by MUSCLE v3.8.31 (Multiple Sequence Comparison by Log-Expectation) [48], and the phylogenetic trees were constructed from full-length protein sequences using Maximum Likelihood approach (IQ-TREE v2.2.0), with substitution models automatically selected and branch support assessed using 1000 bootstrap values. The visualization and modification of phylogenetic trees were performed using iTOL (https://itol.embl.de/upload.cgi, 19 March 2026). Branches with bootstrap values higher than 50 were displayed. GH28 protein sequences of *Setosphaeria turcica* retrieved from the NCBI database (GenBank accessions: XP_008028141.1, XP_008027493.1, XP_008028401.1, and XP_008022078.1) were used as the outgroup.

### 5.5. Synteny Analysis

To explore syntenic relationships, the GH28 proteomes of *B. dothidea* was compared against those of *B. cortices*, *B. qingyuanensis and B. fabicerciana* using BLASTP (*E*-value ≤ 1 × 10^−10^). Gene collinearity between *B. dothidea* and the other *Botryosphaeria* species was then performed using MCScanX v1.0 [49], with the resulting synteny blocks visualized via TBtools v2.096 [50].

### 5.6. Plant Material Preparation and Pathogenicity Assays

Detached branches of two-year-old Chinese hickory (*Carya cathayensis* Sarg.) (cv. ‘Zhelinshan 3’) were used for pathogenicity assays. Healthy branches were collected and maintained under controlled conditions at 25 °C and 75% relative humidity before inoculation. The wild-type *B. dothidea* strain BDLA16-7, three Δ*BdGH28_3* mutants, and corresponding complemented strains were cultured on potato dextrose agar (PDA) at 25 °C. Five-day-old mycelia were used for inoculation. For inoculation, one wound was generated on each detached branch using a sterile knife, and a 1 cm-diameter mycelial plug was placed onto the wounded area. Inoculated branches were maintained under the same conditions. Based on our previous pathogenicity assays using the same host–pathogen system [27], 15 dpi was selected as the endpoint for quantitative assessment because clear differences in lesion development among fungal strains could be readily distinguished at this time point. At 15 dpi, the bark surrounding the inoculation site was carefully removed to expose the underlying necrotic tissue, because the thick bark of Chinese hickory limited direct visualization of lesion development. The necrotic lesion area surrounding each inoculation site was directly measured using ImageGP 2 [51] and recorded in mm^2^. Lesion area at 15 dpi was used as the quantitative measure of fungal virulence. Three independent detached branches were used as biological replicates for each fungal strain at each time point. Because bark removal was destructive and prevented subsequent assessment of the same inoculation site, separate branches were used for different time points.

### 5.7. Expression Analysis of GH28-Encoding Genes of B. dothidea

Based on RNA-Seq data from our previous study [27], significantly differentially expressed GH28-encoding genes were identified during *B. dothidea* infection. Log2-transformed fold changes were calculated to characterize expression patterns, with values ≤ −1 and ≥1 representing down-regulation and up-regulation, respectively. To validate the RNA-seq expression profiles, tissues surrounding the inoculation sites were collected from inoculated Chinese hickory branches at 0, 3, 8, and 15 dpi. Total RNA was extracted using TRIzol reagent (TaKaRa Inc., Dalian, China) and reverse-transcribed into cDNA using the Superscript IV Reverse Transcriptase cDNA synthesis kit. The resulting cDNA was diluted five-fold and used for real-time RT-PCR analysis using SYBR Green chemistry on a Bio-Rad real-time PCR system. The β-tubulin gene of *B. dothidea* (*BDLA_00007187*) was used as the internal reference gene. Gene-specific primers were designed using Primer3 v4.1.0 software (https://primer3.ut.ee/, 12 May 2026) and are provided in Table S3.

### 5.8. Statistical Analysis

Differentially expressed genes (DEGs) were identified from the previously generated RNA-seq data using DESeq2 v4.2. Genes with an absolute log2 fold change (|log2FC|) ≥ 1 and a Benjamini–Hochberg-adjusted *p*-value (FDR) < 0.05 were considered significantly differentially expressed. For qRT-PCR assays, three independent biological replicates were analyzed for each time point, and differences in transcript abundance among time points were evaluated separately for each *BdGH28* gene using one-way analysis of variance (ANOVA) followed by Tukey’s multiple-comparison test. For pathogenicity assays, lesion area (mm^2^) was used as a continuous quantitative measure of lesion development and fungal virulence. Three independent detached branches were used as biological replicates for each fungal strain at each time point, and lesion-area measurements were summarized as mean ± standard deviation (SD). Differences in lesion area among fungal strains were evaluated using one-way ANOVA followed by Tukey’s multiple-comparison test. A *p* < 0.05 was considered statistically significant.

## Data Availability

All data supporting the findings of this study are included in the published article and its Supplementary Materials.

## Supplementary Materials

The following supporting information can be downloaded at https://www.mdpi.com/article/10.3390/plants15162547/s1. Table S1: GH28 peptide sequences of *Botryosphaeriaceae* Species; Table S2: Annotation of BdGH28_3-interacting partners; Table S3: Primers used for qRT-PCR analysis of *B. dothidea* GH28 genes.
